# Stem Cell Secretome for Spinal Cord Repair: Is It More than Just a Random Baseline Set of Factors?

**DOI:** 10.3390/cells10113214

**Published:** 2021-11-18

**Authors:** Krisztián Pajer, Tamás Bellák, Antal Nógrádi

**Affiliations:** Department of Anatomy, Histology and Embryology, Albert Szent-Györgyi Medical School, University of Szeged, H-6724 Szeged, Hungary; pajer.krisztian@med.u-szeged.hu (K.P.); bellak.tamas@med.u-szeged.hu (T.B.)

**Keywords:** spinal cord injury, secretome, lesion-induced secretome, mesenchymal stem cells, neural stem cells, induced pluripotent stem cells, cytokines, neurotrophic factors

## Abstract

Hundreds of thousands of people suffer spinal cord injuries each year. The experimental application of stem cells following spinal cord injury has opened a new era to promote neuroprotection and neuroregeneration of damaged tissue. Currently, there is great interest in the intravenous administration of the secretome produced by mesenchymal stem cells in acute or subacute spinal cord injuries. However, it is important to highlight that undifferentiated neural stem cells and induced pluripotent stem cells are able to adapt to the damaged environment and produce the so-called lesion-induced secretome. This review article focuses on current research related to the secretome and the lesion-induced secretome and their roles in modulating spinal cord injury symptoms and functional recovery, emphasizing different compositions of the lesion-induced secretome in various models of spinal cord injury.

## 1. Incidence, Types and Consequences of Spinal Cord Injury

Spinal cord injury (SCI) is one of the most complicated and devastating neurological conditions, which leads to motor, sensory and vegetative dysfunctions [1,2]. SCI is recognized as a worldwide health problem due to its huge impact on life quality and serious socioeconomic burden. Moreover, the majority of SCI patients suffer from multiple injuries which contribute to associated complications during acute and long-term care [3]. The average lifetime cost of treating an individual with SCI is estimated to be USD 1.5–3.0 million, depending on the location, extent and severity of the injury, as well as the age of the patient [4].

Approximately 90% of SCIs result from traumatic injuries and only 10% of them are caused by diseases (tumours, spondylolisthesis, etc.) acquired at birth or later in life or surgery-related manoeuvres [5]. On a global level, traffic accidents are the most frequent causes of traumatic SCIs, followed by falls from heights, violence (gunshot and stab wounds) and different sport/recreational activities [6]. Falls primarily affect the older population, and their incidence is expected to increase due to the higher average lifetime, especially in highly developed countries [7]. The annual incidence of SCI is estimated at 30 to 70 cases per million population worldwide, resulting in more than half a million new patients every year. Men sustain SCIs approximately four times more often than women, with a peak in the socially most active younger adults [8].

The amount of disability depends on the severity and the level of the injury. Traumatic SCIs can be classified as cervical, thoracic or lumbar injuries. Various studies have demonstrated that SCI occurs in 50% of the cases at the cervical levels, which causes a high mortality rate due to damage of the respiratory motoneurons and close proximity to the medulla oblongata [9]. SCIs can also be distinguished as complete or incomplete, depending on the availability of motor, sensory and vegetative functions distal to the injury. The severity of the trauma is accurately graded by the American Spinal Injury Association (ASIA) impairment scale [10].

## 2. Pathophysiology of Spinal Cord Injury

It is well established that the pathophysiology of SCI can be divided into three overlapping stages: acute, subacute and chronic. During the time course of the SCI, the initial mechanical trauma is followed by a secondary phase which leads to increased dysfunction and cell death over hours to months after the primary insult [11]. It is important to note that the primary phase cannot be prevented, but the outcome of the secondary processes can be managed [12].

In the acute phase of SCI, the direct mechanical force disrupts the structural integrity of the neural tissue, which includes the necrosis of cells, transection of axons and vascular damage [2,13,14]. During this phase haemorrhage, local oedema and neurotransmitter disturbances can also emerge [2,15]. Hypoxia and haemorrhage are both consequences of the disruption of the blood–spinal cord barrier (BSCB), which damage the grey matter due to its high metabolic activity [16].

In the subacute phase, a cascade of secondary events dominates the site of injury, including free radical production, lipid peroxidation and immune-mediated neurotoxicity [2,17]. Glutamate plays a key role in a highly disruptive process known as excitotoxicity, which causes a massive influx of calcium ions into the neurons. This can trigger a series of destructive events, including the production of free radicals, which can attack membranes and other cell components, killing healthy neurons as well as oligodendrocytes [18]. Immune cells invade the injury site and produce proinflammatory cytokines, which are known to lead to apoptosis and affect further spinal segments rostral and caudal to the original damage [19]. The entry of peripheral immune cells is facilitated by increased vascular permeability and disruption of the BSCB.

During the subacute and chronic phases, a centrally located cyst forms in the spinal cord, while hypertrophic astrocytes, along with other cells, secrete inhibitory molecules such as chondroitin sulphate proteoglycans (CSPGs) [15,20]. This process leads to the formation of a glial scar, which represents a physical and a chemical barrier to axonal regeneration [21,22]. Other extracellular matrix proteins such as ephrins are also involved in glial scar formation and inhibit the regeneration of axons [23]. On the other hand, the role of astrogliosis and glial scars can also be beneficial, as they can restore the BSCB, resulting in isolation of the lesion microenvironment [19,24]. Myelin-associated glycoproteins (MAGs), oligodendrocyte-myelin glycoprotein (OMgp) and neurite outgrowth inhibitor-A (NOGO-A) are also upregulated and act on the receptors associated with the Rho-ROCK pathway to stop axonal outgrowth [25]. The onset of the chronic phase can occur months after injury, and this phase continues throughout the patient’s life.

## 3. Immunomodulatory Processes after SCI

Neutrophil granulocytes, resident microglia cells and monocyte-derived macrophages migrate to the site of injury and play a critical role in the degeneration and regeneration processes following SCI.

The rapid activation of immune cells leads to an increase in proinflammatory cytokines, including interleukin-1 (IL-1), interleukin-6 (IL-6) and tumour necrosis factor (TNF)-alpha. Other molecules are also present, such as monocyte chemoattractant protein 1 (MCP-1), matrix metalloproteinase 9 (MMP-9) and stromal cell-derived factor 1 (SDF-1). These soluble factors are involved in further migration of the immune cells into the lesion and in the mediation of glial scar formation [24,26].

Although inflammation has primarily been shown to be neurotoxic, there is plenty of evidence suggesting a neuroprotective role as well. The M1 phenotype of macrophages is considered as the proinflammatory subtype that secretes cytokines and chemokines immediately after the injury, leading to further progression of secondary injury. However, the M2 phenotype is known to be anti-inflammatory and produces the anti-inflammatory cytokine interleukin-10 (IL-10), which leads to regeneration of injured spinal tissues and facilitates wound healing [27,28,29,30,31]. M1 and M2 macrophages coexist at the lesion epicentre during the first week after injury, but only the M1 macrophage can reside up to a month at the lesion site. It is unclear what determines the expression of these macrophage subsets, and their activation appears to be driven by the lesion-related factors present at the time of injury. Myelin debris, as well as considerable TNF-alpha expression, are just some of the factors that reportedly prevent M1-to-M2 conversion [30,31].

Several lines of evidence have suggested that macrophage phenotypes are determined by the microenvironment and can change in response to new stimuli; this adaptivity enables them to contribute to all phases of repair [32].

## 4. Treatment Opportunities for Spinal Cord Injury

At present, the management of SCIs is limited to supporting respiratory function, blood pressure control and surgical decompression [33,34]. Recent studies have shown that methylprednisolone treatment is not effective after injury and is associated with a higher rate of medical complications such as sepsis, respiratory deficiency, durotomy or meningitis [35,36]. In contrast, riluzole, a currently approved drug for the treatment of amyotrophic lateral sclerosis (ALS), has shown effectivity in improving the outcome after SCI [33,36,37].

In general, there are three main strategies for improving the morphological and functional outcomes after SCI: pharmacological intervention, cellular transplantation or forcing the plasticity of the surviving nerve cells. Cell transplantation is one of the most promising possibilities for curing SCI because the stem cells or their derivatives can differentiate into various cell types (neurons, astrocytes, oligodendrocytes), support the survival or regeneration of the host tissue, and can alter the lesion microenvironment via the secretion of bioactive molecules [38,39,40].

In this review, we summarize the effect of factors secreted by grafted mesenchymal, neural and induced pluripotent stem cells that may mediate the immune response, support the preservation of host tissue or induce axonal regeneration.

## 5. Classification of Stem Cells

The stem cells of embryonic and adult organisms are non-specialized cells which go through several steps of specialization. Totipotent stem cells have the highest differentiation potency, which allows them to form both embryonic and extra-embryonic structures. Pluripotent stem cells (PSCs) such as embryonic stem cells (ESCs) are able to form all germ layers, including the ectoderm. Multipotent stem cells, including mesenchymal stem cells (MSCs) and neural stem cells (NSCs), have a limited spectrum of differentiation compared with PSCs and they can specialize only into specific cell lineages [41].

Although many other groups of stem cells are known, we focus here on MSCs, and embryonic and induced pluripotent stem cell-derived NSCs.

## 6. Mesenchymal Stem Cells Used for Spinal Cord Repair

MSCs are multipotent self-renewable cells and are found in various tissues such as bone marrow, adipose tissue, Wharton’s jelly, dental pulp, skin and peripheral blood [42,43,44]. These spindle-shaped cells express various specific surface antigens (CD105+, CD73+, CD90+, CD45-, CD34-, CD14-/CD11b-, CD79α-/CD19-) as established by the Mesenchymal and Tissue Stem Cell Committee of the International Society for Cellular Therapy (ISCT) [45].

Although MSCs form a heterogeneous cell population, they are a good candidate for autologous and allogeneic cell therapies for regenerative medicine. These cells contribute to haematopoiesis and influence bone growth and remodelling [46,47,48]. Under specific conditions, MSCs are able to differentiate into chondroblasts, osteoblasts and adipocytes [49]. Cultured MSCs synthesize and secrete neurotrophic factors and extracellular matrix components including nerve growth factor (NGF), brain-derived neurotrophic factor (BDNF), glial cell line-derived neurotrophic factor (GDNF), ciliary neurotrophic factor (CNTF), Collagen I, Collagen IV, fibronectin and laminin [50]. They are able to differentiate into neurons or astrocytes in vitro and in vivo and adopt the Schwann cell phenotype [51,52].

MSCs have been intensively studied for their potential in restorative approaches for spinal cord repair. Human MSCs grafted into adult immune-suppressed rats following mild, moderate or severe spinal cord contusion injury were able to induce improved functional recovery compared with the controls [53]. Others delivered MSCs into the injured spinal cord to study their fate and possible effects on functional outcome. The grafted MSCs were able to survive for 5 weeks after grafting and induced significantly improved functional recovery compared with the control animals [54]. Deng et al. showed that MSCs grafted intraspinally 2 weeks after injury were able to elicit de novo neurogenesis and functional recovery in rhesus monkeys [55]. While a number of studies have demonstrated the positive effects of MSC transplantation in animal models of SCI, other experiments have shown only negligible functional and morphological recovery [54,56,57,58].

Systemic administration of MSCs has the potential to improve white matter sparing and functional improvement after SCI through the local release of trophic factors. Several studies have shown that intravenous administration of MSCs following SCI had an immunomodulatory effect and proved to be neuroprotective through paracrine production of anti-apoptotic molecules and trophic factors, which promoted neuronal plasticity [59,60,61]. In the case of intravenous administration of MSCs, chemoattractant molecules produced primarily by the damaged tissue may play a role in the migration of the MSCs towards to the injured area. The homing properties of transplanted MSCs can be affected by a number of soluble factors produced by the injured area, including TNF-alpha, vascular endothelial growth factor (VEGF), hepatocyte growth factor (HGF), platelet-derived growth factor (PDGF), fibroblast growth factor (FGF), insulin-like growth factor (IGF), SDF-1, and MCP-1 and -3. These processes are based on the interaction between the surface receptors of the implanted cells and their ligands, involving different signalling pathways [62].

It has been proven that most of the neuroregenerative properties of MSCs in the injured spinal cord are due to the release of a wide range of neurotrophins, cytokines and microvesicles (the so-called secretome), rather than the ability to differentiate into neuronal or glial cells. However, it remains unclear whether these cells are able to alter their secretome expression profile after transplantation, and it is not known how the composition of the secretome changes in different injury models.

## 7. The Secretome Contains Bioactive Molecules

The use of the secretome in regenerative medicine promises a new therapeutic approach. However, one of the pivotal aspects of therapy is its mechanism of action. The different secretory fractions require thorough characterization due to their immunomodulatory and regenerative and/or protective potential. Another important aspect is the possible interaction between the different factors of the secretome and the host tissue, which acts synergistically to enhance the therapeutic effect.

As described above, MSC are able to stimulate, through their secretions, the division and differentiation of different cells and induce immunomodulatory, anti-inflammatory, neuroprotective/neurotrophic and angiogenic effects on the host microenvironment (in the case of SCI) [63]. MSCs are able to secret a variety of soluble anti-inflammatory molecules, including TNF-β1, interleukin-13 (IL-13), interleukin-18 (IL-18) binding protein, CNTF, neurotrophin-3 (NT-3), IL-10 and interleukin-27 (IL-27). In addition, MSCs may affect the host’s cytokine production and thereby stimulate or inhibit the release of anti-inflammatory cytokines (such as interferon-γ and IL-10) [64,65]. It is very likely that these factors play a significant immunoregulatory role that promotes regenerative processes [66]. Vawda et al. have shown, in an elegant study, that the early intravenous administration of the concentrated cell secretome from human umbilical cord matrix cells (HUCMCs) or bone marrow mesenchymal stromal cells (BMSCs) decreased vascular damage but did not improve long-term functional recovery after SCI [63]. Interestingly, the HUCMCs’ concentrated cell secretome was found to be most effective at limiting vascular damage compared with the BMSCs’ secretome.

The secretome often contains the so-called vesicular fractions or extracellular vesicles (EVs), which themselves can produce a therapeutic effect. EVs are involved in cell-to-cell communication and may contain all the signals necessary for successful contact [67]. EVs may contain nucleic acids such as mRNAs, microRNAs and tRNAs that have the potential to target factors involved in immune-related mechanisms [68]. The small size of EVs also offers significant therapeutic benefits, including reduced macrophage phagocytosis and improved extravasation through the injured site. The therapeutic potential of EVs has been investigated in SCI [69,70]. Some studies have provided evidence that intravenous administration of neural progenitor/stem cell-derived EVs significantly reduced neuronal apoptosis, microglia activation and neuroinflammation, thereby promoting functional recovery after SCI in rodents [71,72]. Another report suggested that intrathecal administration of the subventricular zone-derived EVs resulted in remarkable motor recovery by suppressing the formation of the NLRP3 inflammasome complex [73]. Furthermore, EVs were able to modulate the activity of target cells by interacting directly with the cell and to induce angiogenesis, thus decreasing the vascular damage [67,74].

In addition to the facts mentioned above, however, the potential therapeutic mediators of the secretome need to be clarified. The therapeutic targets must be identified and the therapeutic potential of the secretome should be exploited. The therapeutic options offered by the secretome should be examined in the context of trauma or neurodegeneration. The interaction between the molecules and the vesicles, or only among the trophic factors/vesicles, has been demonstrated to be a mediator of the mechanism of action of the secretome.

## 8. Neural Stem Cell Transplantation to Promote the Functional Outcome of the Injured Spinal Cord

There has long been a dogma that neurons are formed only during embryogenesis. Altman and Das were the first to describe the formation of new neurons in the hippocampus of the adult rat brain [75,76]. Neurogenesis was observed in the subventricular zone of the brain and the periventricular zone of spinal cord, which occurs as an extremely limited process in adult life [77,78].

Transplantation of NSCs into a damaged central nervous system (CNS) appears to be a promising therapeutic approach for traumatic spinal cord injuries. However, this requires an appropriate cell dose and cell source. Three main sources can be mentioned: direct isolation of cells from the CNS, differentiation from pluripotent stem cells and trans-differentiation from somatic cells [79].

Several studies have reported that the use of NSCs in the damaged spinal cord could reduce the inflammatory response, promote the replacement of lost neurons and oligodendrocytes, and remyelinate damaged spinal descending/ascending axons, thereby enabling functional recovery [80,81,82]. Previous studies have shown that neuronal stem cells were able to survive after transplantation and differentiate into neuronal or mainly glia-like cells [38,80,82,83,84]. Lu and colleagues transplanted NSCs suspended in a fibrin matrix containing growth factors into a severely injured spinal cord [85]. Graft-derived neurons were able to extend their axons rostro-caudally from the lesion site over a long distance and form new functional synaptic connection with the host neurons. The repeated reassessment experiments by Sharp et al. have shown that NSC grafts were able to fill the lesion cavity, but most grafts did not create a continuous tissue bridge between the rostral and caudal ends of the lesion [86]. Although this study confirmed that grafted NSCs were able to send their axons into the host tissue, it did not confirm the extensive ingrowth of host axons into the transplant or enhanced recovery of motor function in grafted rats.

Grafted NSCs have the ability to differentiate into neurons and form new functional synaptic connections with the host tissue after spinal cord injury but few studies have focused on their capability to release a wide range of factors after grafting [39,82,87]. These studies provided evidence that grafted NSCs secreted a range of neurotrophic factors or cytokines that may also support neural repair [39,82,83,87]. Furthermore, these results raised the possibility of a paracrine mechanism of action, resulting in the close interaction between grafted neural stem cells and the host tissue.

## 9. The Lesion-Induced Secretome Produced by Grafted Stem Cells

### 9.1. The ESC Secretome for Treating SCI

ESCs can produce growth and neurotrophic factors as well as pro- or anti-inflammatory cytokines to support the survival of injured neurons, initiate axonal sparing/regeneration and modulate immune reactions. Because ESCs carry the risk of tumorigenicity, they are usually induced to differentiate into neural progenitor cells (NPCs), which can then be transplanted into the CNS. Brederlau et al. has shown that the rate of teratoma formation depends on the degree of differentiation [88]. Blocking proliferative signalling pathways or sorting the cells before transplantation are the other options for decreasing the risk of tumour formation [89]. The application of ESCs or their differentiated forms into rat, mouse or monkey SCI models significantly improved the morphological and functional outcome of SCI [90,91,92,93,94]. ESCs secrete bioactive molecules such as VEGF, MMP-9 and MCP-1 that enhance the clearance of apoptotic cells and myelin debris, support tissue preservation/regeneration and modulate the immune reaction. They can also produce anti- and proinflammatory molecules which are able to promote the conversion of M1 microglia cells into an M2-like state. This process can effectively modulate the inflammatory responses, leading to a growth-permissive microenvironment after SCI [95,96,97]. The first clinical trial of hESC-derived oligodendrocyte progenitor transplantation approved by the FDA began in 2009. However, Geron Corporation terminated the trial due to financial reasons [97]. Fortunately, renewed funding has allowed the study of these cells in patients with subacute cervical spinal cord injury to restart [98]. While the clinical application of ESCs is promising, their use in SCI is limited due to technical considerations, immune rejection problems and serious ethical issues which have to be solved [96].

### 9.2. Application of NE-GFP-4C Stem Cells

Recently, we initiated a series of studies to explore the hypothesis that grafted NSCs have the ability to adapt the fate of injury. In our experiments, the NE-4C neuroectodermal cell line was used to study its effect on the injured spinal cord [82,87]. NE-GFP-4C cells (ATCC: CRL-2926) are NSCs first described by Madarász et al.; they were isolated from 9-day-old p53-deficient mouse embryo cortexes [99]. NE-GFP-4C cells implanted in 13-day-old embryonic mouse brains were able to integrate and develop into morphologically differentiated neurons [100]. These data suggest that neuroectodermal stem cells may be a promising candidate to be transplanted into the injured spinal cord.

In our series of experiments, NE-GFP-4C stem cells were grafted into different spinal cord injury models and the fate of the stem cells and their interaction with the host tissue were analysed [82,87]. One of the experimental approaches was the lumbar 4 (L4) ventral root avulsion and reimplantation model. This type of injury results in clear-cut motoneuron damage, where only the motoneurons in the affected segment are damaged. Ventral root avulsion initiates the activation of astrocytes and microglial cells, and the extracellular space becomes loaded with excessive amounts of excitotoxic glutamate, which leads to the death of vast majority of motoneurons [101]. Grafting the undifferentiated NE-GFP-4C cells into the affected spinal segment immediately after the avulsion injury produced modulatory anti- and proinflammatory cytokines (IL-1-alpha, IL-6, IL-10, TNF-alpha and macrophage inflammatory protein-1 (MIP-1) alpha at least for 10 days [87], which rescued the injured motoneurons and enabled them to reinnervate the peripheral targets [102]. The host astrocytes and neurons in the treated animals secreted IL-6 and MIP-1 alpha, indicating strong communication between the grafted cells and the host tissue. Based on these results, NE-GFP-4C cells were applied in a spinal cord contusion injury model. Stem cells were grafted 1 week after the contusion injury, and the grafts produced the neurotrophic factor GDNF, the anti-inflammatory cytokine IL-10 and the proinflammatory cytokines IL-6 and MIP-1 alpha in a paracrine fashion for at least 1 week and induced significant morphological and functional recovery compared with injured untreated animals [82]. Application of osmotic pumps filled with neutralizing antibodies against these four factors nearly completely abolished the effect of the graft [82]. It is important to note that NE-GFP-4C cells did not produce any of these factors in vitro. In the first week after grafting in both injury models, only minor differentiation and migration of grafted cells were observed, and this time period proved to be critical for neuroprotection. Interestingly, after the critical period, the majority of the surviving grafted cells differentiated into neurons or astrocytes and lost the eGFP signal. It can be stated that grafted undifferentiated NE-GFP-4C cells have the capacity to adapt to the specific lesion microenvironment, such as segmental motoneuron injury and spinal cord contusion injury, leading to the functional multipotency of stem cells shown by Teng et al. [103,104].

### 9.3. Delivery of Induced Pluripotent Stem Cells

The recent development of induced pluripotent stem cells (iPSCs) can serve as an alternative stem cell source [105,106]. The iPSCs can be isolated from highly accessible somatic cells such as dermal fibroblasts or blood cells by transient overexpression of defined transcription factors, notably Oct3/4, Sox2, Klf4 and c-Myc [105,106]. Other efficacious factor combinations have also been discovered because some of the factors (c-Myc) were associated with oncogenes. iPSCs display similar properties to ESCs, such as their morphology, proliferation capacity, surface antigens expression and gene expression characteristics [107]. The advantage of iPSCs over ESCs is that iPSCs avoid ethical concerns and the transplantation of iPSCs does not generate a significant host immune response [108]. Studies have also shown that iPSCs preserve the epigenetic memory of the cell type of origin, which results in the accumulation of DNA damage [109]. Miura et al. demonstrated great differences in the tumorigenicity and differentiation capacity of iPSCs derived from various origins [110]. Therefore, much care must be taken to minimize that risk using different approaches, including the use of non-viral techniques or more differentiated cells, such as NSCs [108,111,112].

Various iPSC-derived NSCs were grafted into the injured spinal cord, and considerable information has been gained about their survival, proliferation and differentiation capacity, as well as their migration properties [108,113,114,115,116,117]. Tsuji et al. established various mouse iPSC lines by different methods and detailed an evaluation of the cells, including their differentiation potential and tumorigenic activities. “Safe” iPS-derived neurospheres differentiated into all three neural lineages without forming teratomas after SCI. They proved to remyelinate naked axons and supported the regrowth of host serotonergic fibres, thus promoting functional recovery [118]. In another study, Romanyuk et al. showed that the expression of human neurotrophins significantly changed 8 weeks after cell grafting into the injured spinal cord. After human iPSCs-derived NSC grafting, upregulation of the NGF, FGF8 and GDNF genes and downregulation of the VEGFA and NT3 genes were reported [119]. In some cases, iPSC-derived NSCs induced great differences in motor function recovery due to the age of the somatic cell donor [120]. Please modify to Moreover, adult tissue-derived iPSCs were not as safe as embryonic-derived clones and could form a teratoma after implantation into the injured cord [121]. Interestingly, some studies showed very low or no significant effects after iPSC-derived cell transplantation [120,122,123].

As we described above, grafted NE-GFP-4C stem cells were able to adapt to the damaged environment and produce the specific lesion-induced secretome. Consequently, the question arose as to whether grafted undifferentiated iPSCs have the same capacity as undifferentiated NSCs.

Our lab has demonstrated that grafted undifferentiated mouse iPSCs produced neurotrophic factors (NT-4, GDNF) and cytokines (IL-10, MIP-1 alpha) in the first 7 days after grafting in a ventral root avulsion model [124]. Moreover, the injured host tissue expressed various cytokines and neurotrophic factors, including GDNF, IL-10 and MIP-1 alpha, indicating a strong signalling and modulatory process [124]. Accordingly, grafted animals showed significantly better morphological and functional recovery than injured and untreated controls. Human undifferentiated iPSCs grafted into the lesion cavity following spinal cord contusion injury produced various factors, such as the neurotrophic factor GDNF, the anti-inflammatory protein IL-10 and the proinflammatory protein MIP-1 alpha for at least 1 week. hiPSCs induced tissue sparing, the preservation of axons of propriospinal and supraspinal neurons and limited the deposition of CSPGs, which led to better functional improvement [125]. Furthermore, our in vitro results have provided evidence for the secretion of GDNF, TNF-alpha and VEGF by undifferentiated hiPSCs [125].

In case of grafted iPSCs, only minor phenotypic changes were observed within the first week after grafting. Transplanted hiPSCs survived for up to 2 weeks in the injured cord and underwent a relatively fast differentiation process, accompanied by an intense and increasing microglial/macrophage response by the host immune system [125]. These results are in agreement with the microglia/macrophage reaction against grafted mouse iPSCs in the motoneuron injury model. Based on these results, it can be stated that grafted undifferentiated iPSCs also have the ability to interact with the host tissue and induce morphological and functional recovery via a paracrine mechanism.

**Figure 1:**
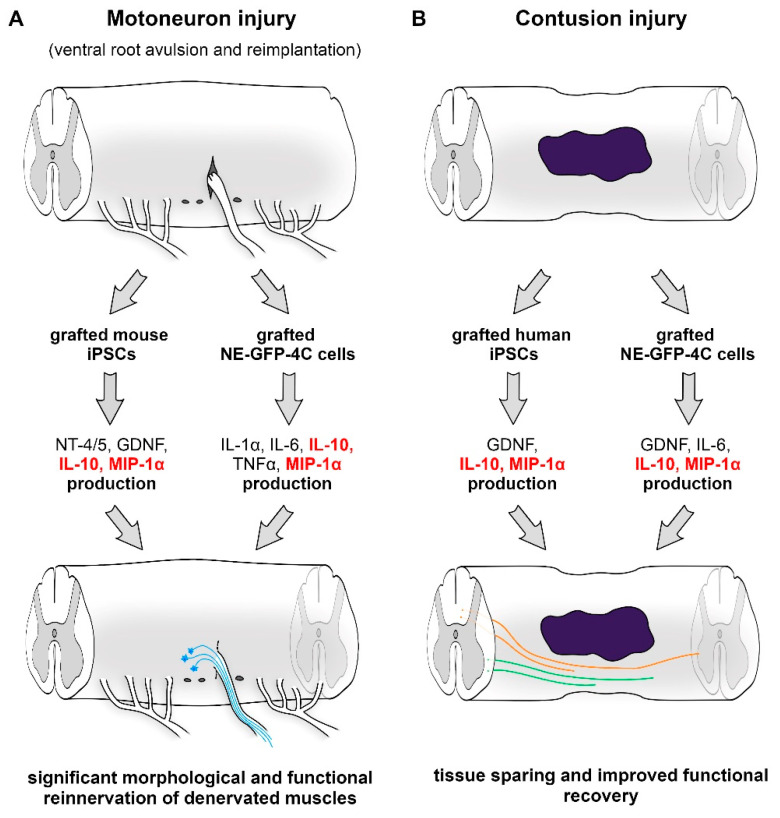
Various of lesion-induced secretome compositions in different spinal cord injury models. Partial overlap (IL-10 and MIP-1a) is observed between the two sets of bioactive molecules. (**A**) Following ventral root avulsion and reimplantation, the grafted cells produced various factors that induced significant functional motor reinnervation of the denervated muscles. (**B**) In a contusion injury model, the grafted cells expressed a set of proteins that significantly contributed to neuroprotection and improved the functional outcome of spinal cord injury.

It is worth noting that NE-GFP-4C stem cells and iPSCs showed different secretome patterns with a partial overlap (IL-10 and MIP-1 alpha) (Figure 1). Nevertheless, in each case, they were able to achieve significant morphological and functional recovery. The soluble factors mentioned above have reverse functions in the pathophysiology of spinal cord injury, but they can act together and mediate the host immune response, induce tissue sparing and support axonal sparing/regeneration.

### 9.4. Effects of Lesion-Induced Secretome Components

IL-10 is an effective anti-inflammatory cytokine and induces a number of signalling cascades which activate the antiapoptotic pathway in neurons [126]. IL-10 administration after SCI promoted the survival of the injured neurons and improved motor recovery [127,128]. The role of the proinflammatory cytokine MIP-1 alpha is controversial, but it can control cytokine expression in the injured CNS and modulate the immune response after SCI [82,87,124,125,129,130].

IL-1 alpha, IL-6 and TNF-alpha are prominent proinflammatory cytokines produced rapidly after spinal cord injury and have overlapping functions in the CNS. They can modulate synaptic plasticity and glutamatergic transmission, and contribute to the survival of injured neurons [131,132]. They can activate various cell signalling pathways, resulting in protection against excitotoxicity-induced cell death and stabilization of calcium homeostasis with increased expression of the calcium-binding protein calbindin. In vitro administration of IL-6 enhanced neurite outgrowth through increased expression of growth-associated genes [133,134]. Trans-neuronal delivery of hyper-interleukin-6 supported the regeneration of the corticospinal and raphespinal tracts, which led to functional recovery after severe spinal cord injury [135].

GDNF has a potent survival effect on injured neurons after spinal cord injury, with a reduction in secondary damage and an improvement in motor function [136,137]. GDNF reportedly promotes axonal growth following spinal cord injury, as proven by several studies [138]. Spinal neurons can also be rescued by GDNF treatment during the acute phase of the injury [139]. GDNF can also increase the survival and neural differentiation of grafted cells, as well as reducing tissue loss [140]. Pre-treatment with neurotrophins caused anti-apoptotic effects in cultured cortical neurons, but increased necrosis [141]. NT-4/5 is able to activate numerous intracellular pathways which have an influence on damaged adult spinal motoneurons [142,143], but the exact role of NT4/5 in the injured spinal cord is not fully understood.

### 9.5. The Fate of Grafted Cells

**Figure 2:**
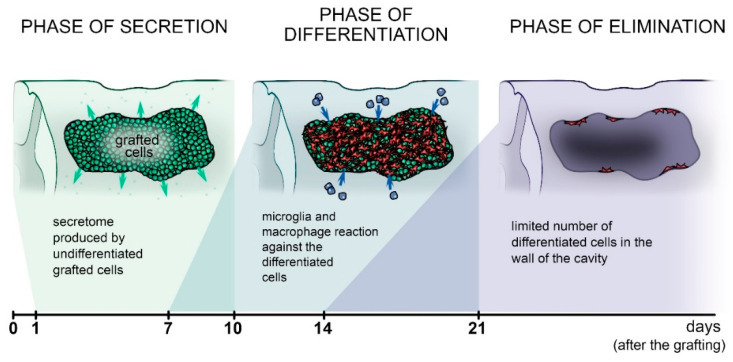
Fate of undifferentiated grafted stem cells in the injured spinal cord. Phase of secretion: Grafted undifferentiated stem cells produce the lesion-induced secretome at least 10 days after grafting. Phase of differentiation: The transplanted stem cells differentiate into neurons and astrocytes, but the microglia/macrophage reaction can be observed against the graft. Phase of elimination: Due to the microglia/macrophage reaction, only a few graft-derived neurons and astrocytes can be detected long-term in the wall of the cavity. Green, undifferentiated transplanted cells; red, graft-derived differentiated cells; blue, microglia/macrophages.

Based on our results, we can identify three overlapping phases after intraspinal grafting of undifferentiated stem cells (Figure 2). First, a secretory phase can be observed, where the grafted undifferentiated stem cells adapt to the injured microenvironment with strong communication with the host tissue. The grafted cells start to express factors, the so-called lesion-induced secretome. Factors produced by grafted cells were coincident with the expression of stage-specific embryonic antigen (SSEA)-1 or -4 markers [82,87,124,125]. This suggests the presence of a narrow time window when grafted cells can produce the lesion-induced secretome. Second, a differentiation phase can be detected, with a time course that is different for each stem cell line. Interestingly, grafted mouse and human iPSCs showed a faster differentiation process than transplanted NE-GFP-4C cells. However, it can be stated for each cell line that the grafted cells started to differentiate mostly at the graft–host interface. At the same time, a very strong immune response begins against the graft. Initially, reactive microglia cells and macrophages merely encircle the graft; a few days later, they invade the grafted area. The third phase is characterized by elimination of the graft due to the strong immunoreaction against the grafted cells. This is an extended phase where slow clearance of the grafted cells is observed. Only a few graft-derived neurons and astrocytes remained in the wall of the cavity in the case of NE-GFP-4C neuroectodermal stem cell transplantation, whereas the undifferentiated iPSCs were fully eliminated by the host’s microglia/macrophage cells [124,125]. Other studies have confirmed that without the use of immunosuppressants or interruption of immunosuppressive treatment, the transplanted undifferentiated stem cells died after a few weeks of survival [144]. The question remains how it could be possible to mimic the beneficial effects of undifferentiated stem cells in an injured cord.

## 10. Possible Applications of the Stem Cell Secretome and the Lesion-Induced Secretome to Spinal Cord Injury

**Figure 3:**
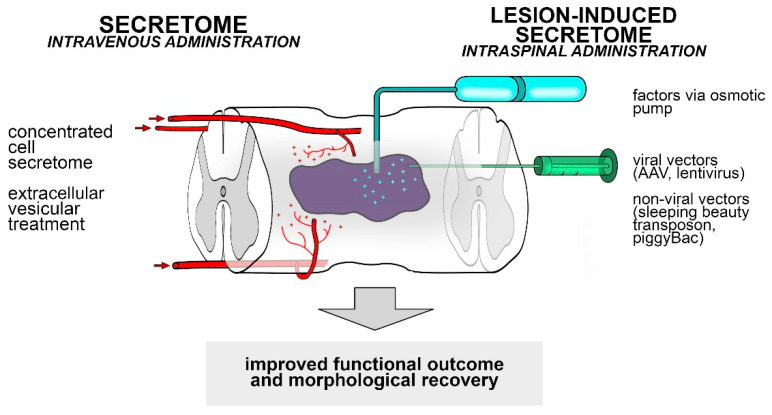
Application of the secretome and lesion-induced secretome via different administration routes. Intravenous administration of the secretome (concentrated cell secretome, extracellular vesicular treatment) produced by MSCs is able to promote neuroregeneration and neuroprotection (**left**). Intraspinal delivery of the lesion-induced secretome to the lesion site by various administration routes such as osmotic pumps and different vectors may be able to induce axonal regeneration and neuroprotection (**right**).

Undifferentiated neural stem cells transplanted into a damaged CNS were able to produce a different lesion-induced secretome composition. The question arises whether the application of these secretome compositions in the injured spinal cord is able to initiate neuroprotective and regenerative processes and thus lead to morphological and functional recovery. To test this hypothesis, we need experimental approaches that allow application of the (lesion-induced) secretome following spinal cord injury (Figure 3).

Intravenous administration of concentrated cell secretome shows storage stability and decreases vascular damage following spinal cord injury [63]. Systemic administration of EVs is also a promising strategy for inducing neuroprotection and neuroregeneration, and decreasing secondary damage after the injury (Figure 3).

In case of intraspinal application of the lesion-induced secretome, a traditional strategy could be to use osmotic pumps that deliver the desired factors to the damaged environment. However, this approach is considered quite invasive because the osmotic pumps can clog and require surgery for removal. Another solution is to use virus-derived vectors that allow the intraspinal application of cytokines and neurotrophic factors. Adeno-associated virus (AVV) vectors have been successfully translated to the clinic for conditions such as inherited retinal disorders or haemophilia B [145]. The AVV vector is a very effective gene transfer system for delivering therapeutic gene products to the injured spinal cord [146]. In other studies, lentiviruses have been used as polycistronic vectors to deliver the genes of multiple therapeutic proteins [147], thus enhancing axonal growth and increasing functional recovery after spinal cord injury. Non-viral strategies, such as plasmids, minicircles, nanoparticles, piggyBac or Sleeping Beauty transposons have the ability to deliver DNA or RNA to a variety of cells through transduction [148]. These vectors allow ex vivo gene delivery into a selected cell population. Carrier cells transfected with Sleeping Beauty transposons or piggyBac may be able to express the proteins transiently, thereby providing a permanent effect. Intraspinal application of these systems would lead to successful delivery of a set of biomolecules into the injured spinal cord and may be able to mimic the beneficial effects of the lesion-induced secretome produced by grafted cells.

## 11. Conclusions

Intravenously administered sectretome-based therapy is a minimally invasive method to target various organs and several pathologies after traumatic SCI. It is important to note that while MSCs are likely to produce secretomes in vitro that are used therapeutically, undifferentiated transplanted NSCs or iPSCs are able to adapt to the injured environment and produce the composition of a given lesion-induced secretome. However, further studies are necessary to prove the positive effects of the identified factors used intraspinally.

Based on our earlier studies it can be stated that the grafted undifferentiated cells first enter a secretory phase and produce a set of factors; then, 5–10 days later, they enter a differentiation phase, losing their secretory function. A few days later, the immune response begins against the differentiated graft-derived stem cells (phase of elimination). In the secretory phase, the secretome composition produced by the grafted cells often contains inflammatory cytokines that have a negative effect on the peripheral immune system. Nevertheless, these inflammatory cytokines, supplemented with neurotrophic factors, form a complex mixture that can significantly promote functional and morphological recovery. The common points of the lesion-induced secretomes identified in each experiment are IL-10 and MIP-1 alpha. In addition to these factors, proinflammatory or neurotrophic factors were produced by the grafted cells, and significant morphological and functional improvements were observed in all cases.

Although the clinical application of undifferentiated stem cell transplantation is not feasible, under experimental conditions, it is possible to graft these cells into the damaged CNS. Due to the factors produced by grafted cells during adaptation to the damaged environment, significant morphological and functional improvement can be induced. The identification and local application of these unique factors may open new avenues for treatment strategies of the injured CNS, and the acute or subacute administration of the secretome or the lesion-induced secretome could conceivably be combined with a later administration of pharmaceuticals to decrease secondary injury.
